# Local Weather Types (LWTs) associated with Urban Heat Islands (UHIs) and hot days in the Grenoble area, France

**DOI:** 10.1371/journal.pone.0339189

**Published:** 2025-12-26

**Authors:** Sandra Chantal Rome, Yingting Wang, Sylvain J. Bigot, Xavier Foissard, Julia Hidalgo Rodriguez

**Affiliations:** 1 Université Grenoble Alpes, CNRS, INRAE, IRD, Grenoble INP, Institut des Géosciences de l’Environnement (IGE), Grenoble, France; 2 Laboratoire Interdisciplinaire Solidarités Sociétés Territoires (LISST), Université de Toulouse, CNRS, Toulouse, France; Institute of Oceanology Chinese Academy of Sciences, CHINA

## Abstract

Urban heat extremes are intensified by both global warming and the urban heat island (UHI) effect, particularly in summer. This study investigates the meteorological conditions driving extreme summer temperatures in Grenoble (French Prealps), a city with complex topography. The objectives are threefold: 1/ Assess the performance of the local weather types (LWTs) classification method in this context; 2/ Adapt and optimizing the method for topographically and climatically heterogeneous environments and 3/ Identify LWTs associated with extreme heat events—heatwaves, tropical nights and UHIs—for operational used by local authorities. Daily meteorological data (precipitation, temperature range, wind speed, wind direction and specific humidity), which are utilized for classification, are sourced from: (i) ERA5 reanalysis at the nearest the rural grid point (1960–2001 and 2002–2022), compensating for the lack of long-term representative data for the area; and (ii) a dense urban observation network (2019–2022). In this study, in order to better represent the complexity of meteorological conditions due to the incised topography, the original LWT method is modified. Methodological adjustments include discretizing precipitation into three categories and excluding wind direction, shown to have negligible influence due to topographic constraints. Results demonstrate that the modified LWT method effectively captures local meteorological variability and is transferable to similar contexts. LWTs linked to extreme heat events in Grenoble are characterized by high diurnal temperature ranges, dry conditions, elevated specific humidity, and low wind speeds.

## Introduction

Anthropogenic greenhouse gas emissions have unequivocally driven global warming, with global surface temperature currently 1.1 °C above pre-industrial levels [1]. One of the most critical consequences of this warming is the increased occurrence, intensity, and duration of extreme heat events, particularly heatwaves, which have intensified since the 1950s at both global and regional scales [2,3] and in many regions worldwide [4–7]. Projections indicate that each additional 0.5 °C of warming will further amplify the severity and frequency of these extremes [8], with heatwaves expected to occur earlier in the season [9–11] under continued warming. In 2023, global mean temperature reached a record 14.98 °C, underscoring the urgency of anticipating the impacts of continued warming, which is on track to reach 1.5 °C by 2033 if current trends persist [12].

The rising frequency of extreme heat events poses major threats to public health [13,14] and ecosystems. Numerous studies have established strong links between high temperatures and increased morbidity and mortality, particularly among vulnerable populations such as the elderly, individuals with pre-existing cardiovascular or respiratory conditions, pregnant women, and infants [15–24]. Between 2014 and 2022, nearly 33,000 heat-related deaths were recorded in France, with two-thirds involving individuals over 75 years of age [25–28]. Similarly, heat extremes are associated with poor prenatal health, particularly low birth weight and premature birth in newborns [29–31], in particular for female babies [32]. Heat also impairs work capacity and cognitive function, due to physiological stress, dehydration, and reduced muscular performance [33,34].

France has already experienced a 1.7 °C rise in average air temperature since 1900, exceeding the + 1.5 °C target set by the Paris Agreement [35]. Urban areas, where three-quarters of the French population reside, are further affected by the Urban Heat Island (UHI) effect—an intermittent meteorological phenomenon, mainly nocturnal, characterized by a positive temperature differential between urban and suburban areas due to the high percentage of impermeable surfaces. While the work of Luke Howard [36] was at the origin of urban climate studies, those of Timothy Oke [37–41] on urban energy and water balances that more specifically initiated UHI studies. The UHI effect intensifies summer heat and exacerbates thermal discomfort, particularly in topographically constrained cities like Grenoble.

The ability to anticipate and respond to climate events is one of the keys to improving public health in the face of increasing extremes of heat, including in France. We need to anticipate the probability and occurrence of high-impact events. Whatever the case, extreme heat is both a challenge and a management concern for municipal authorities, whose aging population is considered highly vulnerable.

Understanding the atmospheric drivers of extreme heat is essential for improving public health preparedness and guiding urban planning. Weather type classifications provide a concrete framework for characterizing near-surface meteorological conditions. The notion of weather type described by Durand-Dastès [42] was introduced into climatology studies with the aim of providing a close-up view of everyday reality, giving a concrete description of local climates, avoiding in particular the drawbacks of “separative” statistics, and providing elements of climate explanation, or “explanatory description”. A weather type groups together all the values taken in a given space and over a given period by the variables describing the state of the atmosphere near the ground (rainfall, temperature, wind, etc.). This notion is to be distinguished from atmospheric circulation types at high altitude also called Weather Regimes or Weather Types Classifications (WTCs) that refer to large-scale circulation patterns; depending on the application, WTCs may also be related to near-surface local variables [43].

Two main methods of classifying weather types have been recently applied to the French territory. The first classification, proposed for studying the UHI effect [44–46], is composed of four meteorological variables: cloudiness, temperature, precipitation, and wind, with a fixed number of classes (up to 64 possible combinations). However, this weather type classification method is limited by the already predetermined classes.

The second method uses local weather situations – referred to here as “sensitive weather types”- called “Local Weather Types” (LWTs). Classifying can help implement climate change mitigation and adaptation policies, while also facilitating the analysis and communication of climate information to local decision makers. In this study, an LWT refers to the description of the atmospheric situation directly stemming from the analysis of climatic data from the atmospheric boundary layer [47]. The method applies the Partitioning Around Medoids (PAM) algorithm [48], using Gower distance to quantify dissimilarities among five daily atmospheric variables. This approach is useful for the technical needs of climate modeling and the management of urban microclimates by local authorities [47,49,50].

Given the increasingly high summer temperatures and the additional UHI effect, the aim of this work is threefold: 1/ to evaluate the applicability of the LWT classification method to Grenoble (France), using available ERA5 reanalysis data; 2/ to improve and optimize the LWT approach to account for the city’s complex topography; and 3/ to identify the LWTs responsible for very high temperatures or involved in extreme summer heat and heatwaves in Grenoble. Despite previous studies investigating the link between UHI and local weather types (LWTs) across 45 French conurbations, Grenoble was excluded due to its complex orography [51]. Evaluating the Grenoble case will enable local authorities to better anticipate heat extremes (operational goal).

## Study area

**Fig 1:**
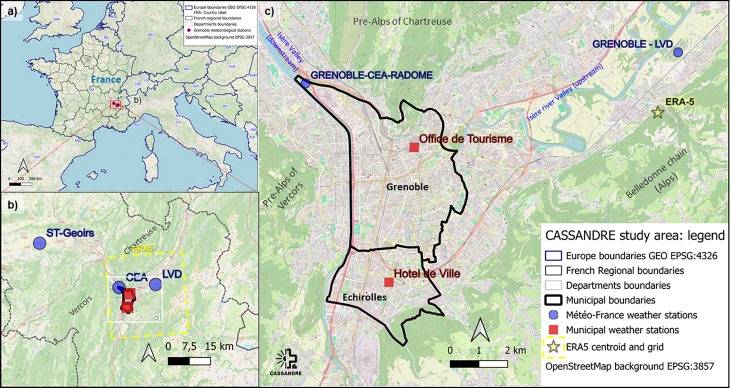
Location of the study area. (a) situated at the bottom of an Alpine valley, (b) specifically the Isère river valley **(b & c)**. The dotted yellow square indicates the ERA5 grid corresponding to the Grenoble rural area, and the yellow star indicates the grid centroid **(b)**. **(c)** The Grenoble-CEA-Radome (CEA) station is the official meteorological station since 2019, not used in this paper due to insufficient available data length over time. Grenoble Saint-Geoirs and Le Versoud stations are abbreviated to St-Geoirs and LVD respectively. Contains information from OpenStreetMap and OpenStreetMap Foundation, which is made available under the Open Database License.

The Grenoble conurbation is located in the southeast quarter of France, in the Auvergne-Rhône-Alpes region. The main city, which is the capital of the Isère department, is located on a glacial trough—a flat-bottomed valley—at the junction of three valleys forming a “Y” shape, nestled between the pre-Alpine ranges of the Chartreuse to the north, the Vercors to the west, and the Alpine massif of Belledonne to the east [52,53] This topographical configuration, combined with valley-floor conditions and thermally induced breezes, significantly influences the region’s marginal mountain climate. Fig 1a and 1b respectively illustrate the general location of the study and the contrasting topographical context. Fig 1c displays the towns of Grenoble and Échirolles studied in the ADEME-funded CASSANDRE project (https://www.ige-grenoble.fr/CASSANDRE). It also shows the location of the meteorological stations used for studying LWTs in the Grenoble area.

The climate of the Grenoble region lies at the crossroads between Atlantic oceanic influences from the west, Mediterranean influences from the south, and the contrasting topography of the glacial troughs. Grenoble’s climate, defined on the basis of the Grenoble Saint-Geoirs (1971–2000) and Grenoble Le Versoud (2000–2020) stations (Figs 1a and 1c), belongs to the Cfb climate type (according to Köppen’s classification), *i.e.,* temperate with moderately hot summers and no dry season, otherwise classified as “temperate oceanic mountain margins” climate type according to Joly *et al*. [54].

**Fig 2:**
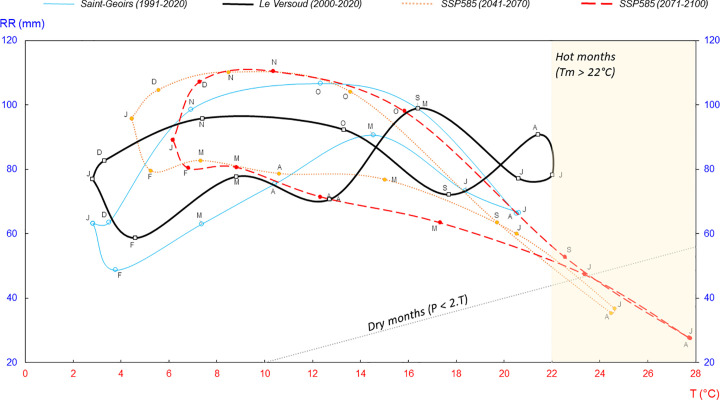
Observed and Projected Climograms for the Grenoble Area. Average climograms from two weather stations in the Grenoble area (Saint-Geoirs, 1991–2020; Le Versoud, 2000–2020) are compared with those derived from CMIP6 projections (ensemble-mean of the SSP5-8.5 scenario) for the periods 2041–2070 and 2071–2100. Model outputs correspond to the grid point associated with Grenoble (data extracted from Climate Explorer). T = temperature (°C); P = precipitation (mm). The dotted line represents the dry-month threshold (P < 2·T). The yellow shading indicates months with mean temperature (Tm) > 22 °C.

The climographs (Fig 2) (based on the analysis of monthly mean temperature values on the x-axis and precipitation on the y-axis) compare the monthly rainfall-temperature variability at two weather stations in the Grenoble region (Saint-Geoirs 1991–2020 and Le Versoud 2000–2020) with future projections calculated from the CMIP6 (*i.e.,* the mean of ensemble means) under the SSP5–8.5 scenario, for the periods 2041–2070 and 2071–2100 (the grid point corresponds to Grenoble; data taken from the KNMI Climate Explorer (https://climexp.knmi.nl/start.cgi). The Saint-Geoirs station is located 38 km northwest of the city and has a long study period; its orographic setting at 384 m in altitude and under the influence of westerly winds, differs from that of the city. The Le Versoud station, located 12 km to the northeast, is considered as the rural reference station for comparison with the urban area.

The average local climate has warmed by +0.8 °C at Grenoble Le Versoud from 2002 to 2022 (not shown) mainly due to maximum temperatures (Tm) (+1.5 °C). It is set to warm further according to future projections; the summer months are likely to be hot (Tm > 22 °C) in the medium term (2041–2070), and even very hot for July and August by the end of the century, averaging over 24 °C (Fig 2). Given these projections, the climate type should soon change from Cfb to Cfa according to Köppen’s classification.

Although a high spatial density temperature dataset in the Grenoble alpine valley (France) exists [53], it has only been in existence since 2019 and for urban heat island investigation and climate services. Given the lack of representative data for the urban climate description over the long term, the use of ERA5 reanalysis offers a pertinent substitute.

## Data and methods

### Types of extreme temperatures

Three types of thermal extremes are considered in the LWT-based analysis: UHI, heatwave and other hot days and nights.

1. **The Urban Heat Island (UHI)**: The UHI phenomenon refers to elevated air temperatures in urban centers compared with the surrounding rural areas [55]. UHI events are identified when the nighttime temperature difference between the urban core (“Office de Tourisme” station for Grenoble and “Hôtel de Ville” station for Échirolles, see Fig 1) and the rural periphery (“Grenoble Le Versoud” station) exceeds 3°C (*i.e.,* ΔTn ≥ 3°C) under low wind conditions at the time of measurement (< 2 m/s), following a method adapted from Foissard *et al.* (56). UHI intensity is categorized as: “none or negligible” (< 3 °C), “moderate” (3 °C to 5 °C), or “high” (> 5 °C). Stronger UHI intensities are associated with increased health risks, particularly for vulnerable populations such as the elderly and very young children [21,56].
2. **Heatwaves:** Although no universally accepted definition exists, this study adopts the criteria derived from the Heat and Health Watch Warning System [57] implemented in France in 2004. This system is based on retrospective analysis of meteorological and health data across fourteen pilot cities. Among seven tested biometeorological indicators, the selected one combines daily minimum and maximum temperatures exceeding specific thresholds for at least three consecutive days. These thresholds, derived from mortality data, are regionally calibrated. For the Isère department, a “biometeorological” heatwave is defined as a period during which the minimum temperature (Tn) exceeds 19 °C and the maximum temperature (Tx) exceeds 34 °C for at least three consecutive days [58,59], aligning with standard national definitions [60]. These thresholds consider the effects on the human body, hence the term “biometeorological.”
3. **Hots days and tropical nights**: Daily maximum temperatures (Tx) are classified into four categories based on intensity: cool days (Tx < 25 °C), warm days (25 °C < Tx < 30 °C), hot days (30 °C < Tx < 35 °C) and very hot days (Tx > 35 °C). Tropical nights are defined as those with Tn exceeding 20 °C. These events are analyzed in relation to the identified LWTs.

### Local Weather Types (LWT): original method

#### Study objective and classification method.

This study employs the LWT classification developed by Hidalgo and Jougla [47] designed specifically for local urban contexts. The LWT method is based on Partitioning Around Medoids (PAM) and Gower distance statistical algorithms using five meteorological variables (temperature and specific humidity at 2 m, precipitation rate, wind direction, and wind strength). Each situation has its own local weather pattern or “sensitive weather,” specific meteorological phenomena with meteorological variables expressed in a similar near-surface meteorological, such as rainy, windy, sunny or cold weather. Sensitive weather depends on the season and geographical location. This definition of LWT allows it to represent the plural nature of weather situations characteristic of a location, as in French cities [61]. This classification is thus objective and not predefined.

### Atmospheric variables

The five daily variables used include:

1. Diurnal thermal amplitude (dT, °C), calculated as a difference between daily maximum (Tx) and minimum (Tn) temperature values (dT = Tx – Tn),
2. Specific humidity (*q,* g kg-^1^),
3. Precipitation (RR, mm),
4. Wind speed (FF, m s-^1^),
5. Wind direction (DD) categorized into four quadrants (1–90°; 91–180°; 181–270°; 271–360°).

The wind direction is treated as a categorical variable, whereas the others are continuous.

### Meteorological data sources

Three datasets are used:

1. **Météo-France Stations:** Non-urban reference data are drawn from Grenoble-Saint-Geoirs and Grenoble Le Versoud (Fig 1). Grenoble-Saint-Geoirs, located 37 km northwest of Grenoble as the crow flies, at 384 m elevation, is excluded due to its distinct topography, higher elevation, and differing wind exposure, making it unrepresentative of urban conditions. Despite offering the region’s longest continuous meteorological record, it is unsuitable for rural–urban comparisons [52]. By contrast, the Grenoble Le Versoud station, located 12 km east-northeast of Grenoble at 225 m on the valley floor—provides a more comparable setting. Although its data series is shorter and lacks humidity data after October 2021, it is retained as the rural reference station for calculating urban–rural thermal differentials.
2. **Urban Observational Data:** Daily data (July 23, 2019 to December 31, 2022) from two urban stations located in the core of the urban area (Grenoble “Office de Tourisme” and Échirolles “Hôtel de Ville” (Fig 1)) are used for UHI intensity analysis during three summers (2020–2022) and comparison with ERA5 reanalysis to assess their quality. These stations have been set up by municipalities to serve as urban reference points and represent the weather and climate variations experienced by their populations. This makes it possible to anticipate actions recommended in order to reduce urban overheating. These stations are part of a dense intra-urban monitoring network [53].
3. **ERA5 Reanalysis:** ERA5 data from the European Centre for Medium-Range Weather Forecasts (ECMWF) (available since 1980 at 0.25° hourly resolution) are used for 2019–2022 (short-term) and 2002–2022 (long-term) to run the LWT algorithm [62]. The grid centroid aligns with the Le Versoud station (LVD in Fig 1c).

### Assessment of data representativeness

**Fig 3:**
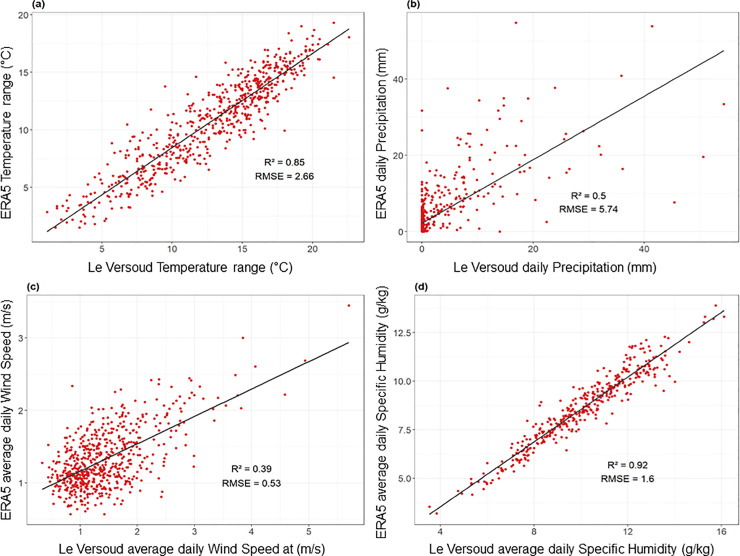
Daily Observations vs. ERA5 Reanalysis at Le Versoud. Scatterplots compare daily values of (a) thermal amplitude, (b) precipitation, (c) wind speed (May–September 2019–2022), and (d) specific humidity (May–September 2019–2021) measured at the Le Versoud meteorological station with the corresponding ERA5 grid-cell data over the same periods. Each point represents one day. The solid line indicates the linear regression between station observations and ERA5 estimates. Specific humidity measurements at Le Versoud have been discontinued since October 2021.

ERA5 reanalysis is assessed against Le Versoud data using coefficients of determination (R²) and Root Mean Square Error (RMSE). Results indicate strong agreement for specific humidity (R² = 0.92), good for thermal amplitude (R² = 0.85), moderate for precipitation (R² = 0.5), and weak for wind speed (R² = 0.39) (Fig 3).

### Adapted method: input parameter modifications

**Fig 4:**
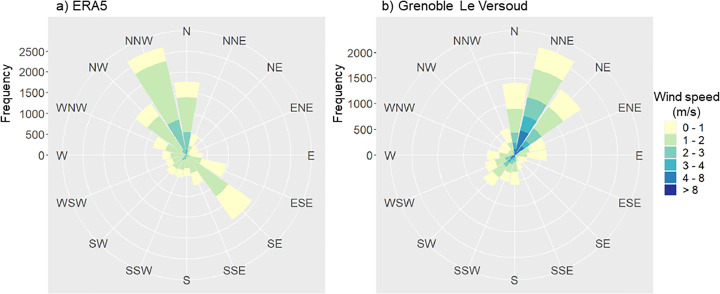
Hourly wind roses from ERA5 and Le Versoud. Hourly wind roses for the period May–September 2019–2022 (14 688 days) derived from **(a)** ERA5 reanalysis and (b) observations at the Le Versoud meteorological station.

The rural station originally used in the LWT method (Saint-Geoirs) does not adequately represent the wind characteristics of Grenoble’s urban environment. While prevailing winds at the rural site are predominantly westerly (Fig 4a), urban observations at the LVD station indicate mainly ENE winds (Fig 4b). Similarly, ERA5 reanalysis show notable discrepancies, with wind roses differing substantially from Le Versoud in both speed (higher at Versoud) and direction (Fig 4). Given the complex topography of alpine valleys, the poor correlation of wind direction, and its limited contribution (as reflected by the low RMSE), this parameter was excluded from the adapted classification. By contrast, wind speed plays a more significant role in the development of UHIs. Known ERA5 biases, such as underestimation of precipitation and cooling, are corrected using a multivariate quantile mapping method [63], which reduces residual biases to below 0.1 K for temperature and −1.5% for relative humidity.

Precipitation, wind, cloud cover, and fog can attenuate UHI effects, depending on its intensity and duration [64]. The formation of cloud and fog are reducing due to urban temperature increase [65]. With little or no precipitation, clear night-time (radiative night), and a stable atmosphere, the UHI will be low. With intense precipitation, cloud cover, and relatively high humidity, the absorption of heat stored at the surface is greater, which can lead to a significant reduction in the UHI.

### The adapted method discretizes precipitation into three categories, based on thresholds that may or may not generate UHIs

1. Dry days (< 1 mm),
2. Moderate rainfall (1–15 mm)
3. Heavy rainfall (≥15 mm) considered exceptional, based on the 90th percentile of daily values (May – September 2002–2022 = 13.45 mm).

The classification focuses on nocturnal UHI, modulated by precipitation intensity. Specific humidity (*q*) is replaced by relative humidity (RH), as RH is more commonly used and directly reflects temperature-dependent moisture content. Furthermore, only 80% of the days during the relevant seasons (closest to each LWT centroid) are used to avoid extreme outliers and ensure representative interpretation.

In summary, the five original variables are reduced to four: diurnal thermal amplitude, relative humidity, wind speed, and categorized precipitation. Wind direction is excluded, and precipitation is discretized (Table 1) by the need to identifying the days/nights prone to nocturnal UHI formation; no matter how much rain there is, as soon as it rains, the UHI fades and even disappears. Sensitivity analyses confirm that excluding wind direction and categorizing precipitation has negligible impact on classification performance.

**Table 1:** Atmospheric parameters: original vs. adapted LWT.

Parameter	Original Method	Adapted Method
Thermal Amplitude	dT (°C)	Same
Humidity	Specific humidity (q, g·kg ⁻ ¹)	Relative humidity (%)
Wind Speed	Daily average (FF, m·s ⁻ ¹)	Same
Wind Direction	4 quadrants	Removed
Precipitation	Continuous values (mm)	3 classes: < 1 mm, 1–15 mm, > 15 mm

### Reduction of classes

Previous LWT classifications [47], identify 7–12 optimal clusters among 45 French cities [50]; with Grenoble often excluded due to its complex topography [51]. Here, hourly RMSE between reconstructed and observed time series is used to select the optimal number of LWTs [47,50]. Although RMSE is lowest for 12 clusters, the difference is minimal (< 0.05) (figure not shown).

Given the study’s focus on summer UHI, the number of clusters is reduced to seven to facilitate interpretation and reduce redundancy. This reduction, combined with simplified input parameters, enables clearer differentiation of meteorological conditions without compromising analytical resolution.

Ultimately, seven LWTs are retained, allowing assessment of how daily weather types relate to thermal extremes: UHI intensity, heatwaves, hot/very hot days, and tropical nights.

## Results

### Identification of LWTs

The LWT classification was applied following the method adapted from [47], using four parameters instead of five, based on ERA-5 daily data (2002–2022) for the grid corresponding to Grenoble.

**Fig 5:**
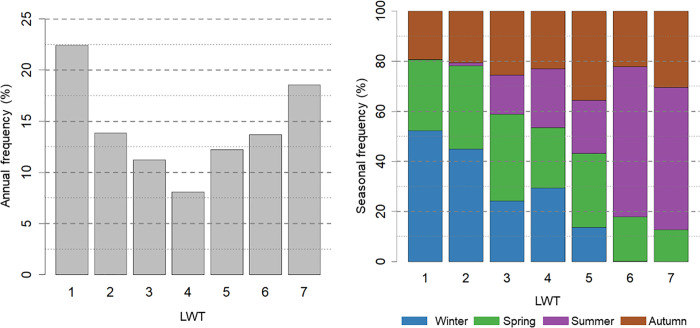
Distribution of the seven LWT in Grenoble area; left, frequency distribution; right, declination by season.

Results indicate a heterogeneous distribution of days across the seven LWTs (Fig 5a). LWT 1 is the most frequent, accounting 22.5% of days, whereas LWT 4 is the lest frequent (approximately 8%). LWT 7 is the second most frequent (18,5%).

At the seasonal scale (Fig 5b, Table 2), LWTs 6 and 7 dominate summer weather conditions, representing over 50% of summer days. LWT 7 typically corresponds to dry days characterized by high diurnal temperature range, low wind speeds, and elevated specific humidity —conditions conducive to heat events, including UHIs and heatwaves. LWT 6 (13.6%), the third most frequent overall, also corresponds to other hot weather situations, often occurring during heatwaves, though not exclusively.

**Table 2:** Average values and frequency of classification variables (dT, RR, q, FF).

LWT	LWT 1	LWT 2	LWT 3	LWT 4	LWT 5	LWT 6	LWT 7
**dT (°C)**	10.71	6.17	6.66	5.59	9.31	9.53	13.33
**RR (mm)**	0.12	5.84	5.68	23.28	0.25	5.5	0.18
**Q (g/kg)**	3.33	4.32	5.41	6.47	5.72	9.36	8.6
**FF (m/s)**	0.78	0.82	1.77	1	1.58	0.74	0.72
**Frequency (%)**	22.43	13.85	11.23	8.07	12.22	13.65	18.54

Winter and spring are mainly associated with LWTs 1 and 2, which correspond to cold weather conditions; These are not further analyzed in this study.

LWTs 3, 4 and 5 encompass a range of transitional conditions, with LWT 3 being more representative of spring and LWT 5 of autumn.

Some LWT are not represented in certain seasons, or only very slightly. For example, LWTs 1 and 2 have few days in summer, indicating that the classification assigned mainly cool to cold winter days, as well as spring days, to these two categories. Similarly, LWTs 6 and 7 are completely absent in winter, as they combine hot summer and autumn days.

**Fig 6:**
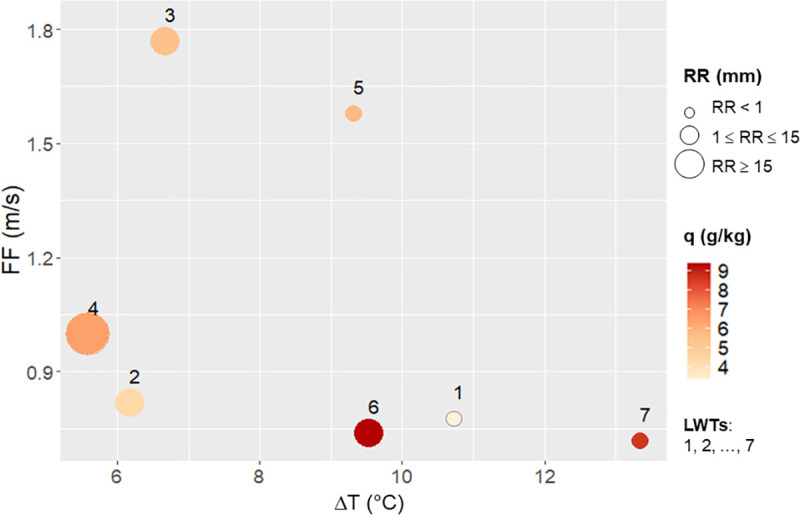
Distribution of LWTs in the Grenoble area according to their average characteristics (dT, FF, RR and q). Digits 1 to 7 indicate the LWT number.

Fig 6 shows the distribution of LWTs along two axes determined by the average of daily thermal amplitude and wind strength. A third dimension is added by considering the average daily precipitation, and a fourth dimension by considering the average specific humidity.

To complement Fig 6, Table 2 shows the mean values of the classification variables. Of all the LWTs, LWT 3 has the highest average wind speed (nearly 1.8 m/s). LWT 7 shows days with a wide diurnal temperature range (dT) (average of dT = 13.3 °C), low specific humidity, no precipitation, and little or no wind. LWT 7 is more characteristic of UHI situations than type LWT 6 which is characteristic of rainy summer days. Only LWT 4 corresponds to the high precipitation category, with an average daily precipitation of 23.3 mm (Table 2).

Table 3 summarizes the characteristics of LWTs, divided into different groups according to thermal amplitude, wind speed, and precipitation intensity in order to distinguish each LWT and better understand their specificities.

**Table 3:** LWTs Classes: thermal and precipitation characteristics (“NF” = absent).

Characteristics	No rain (<1 mm)		Light rainfall ([1–15[mm)	Heavy rainfall (>15 mm)
High temperature range, low wind intensity	**LWT 1** Low temperatures, especially in winter	**LWT 7** Warm and dry summer days	**LWT 6** Hot and rainy summer days	“*NF”*
Low temperature range, low wind intensity	*“NF”*	*“NF”*	**LWT 2** Mainly winter days	**LWT 4** Rainy days
Low temperature range, strong wind	**LWT 5** Mild days, summer temperatures up to 25°C		**LWT 3** Cool & windy days	*“NF”*

It seems clear that LWTs 6 and 7 come into play in situations of very hot weather, with or without UHI, and that LWTs 1 and 7 are associated with winter (low or absent wind and high daily thermal range). LWTs characteristic of hot summers associated with UHIs will be studied in particular.

### Typical summer LWTs

**Fig 7:**
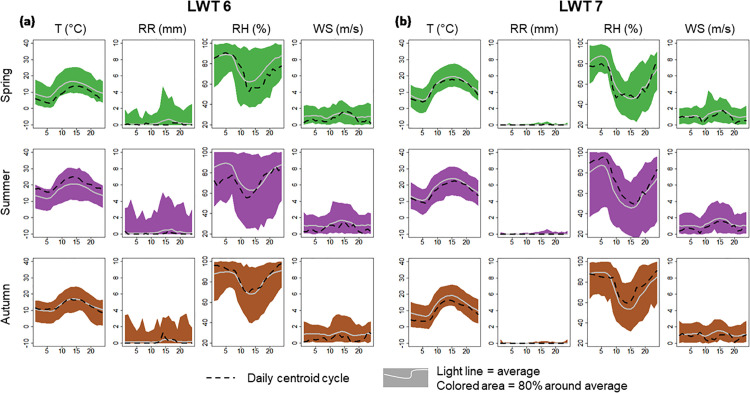
Daily Cycles of LWT-6 and LWT-7 in the Grenoble ERA5 Grid. Daily cycles of air temperature **(T)**, rainfall (RR), relative humidity (RH), and wind speed (WS) for LWT-6 and LWT-7, computed from the ERA5 grid cell over Grenoble for the 2022–2022 period. The solid line represents the mean daily cycle, and the plume corresponds to the range between the minimum and maximum values within the central 80% of the distribution. The dotted black line represents the centroid’s daily cycle. Winter is not shown because too few winter days are associated with these “hot-situation” LWTs.

Fig 7 shows the average daily cycle of air temperature, precipitation, humidity and wind speed for LWTs 6 and 7 which are common summer weather types; They present respectively 60% and 50% of summer days. The difference between the two types is marked by the presence of precipitation and high relative humidity in LWT 6 and, in contrast, quasi-dry conditions and a very high diurnal thermal amplitude in LWT type 7. LWT 6 is marked by mild (Tx ≈ 20 °C) to hot (Tx ≈ 25 °C) summer temperatures and a rather high thermal amplitude (dT = 10 °C). LWT 7 features warm (Tx ≈ 25–30 °C) to very warm (Tx ≈ 35 °C) summer temperatures and the highest thermal range (dT = 13.5 °C).

Given the generally cold bias of ERA5 temperature data, temperatures expected to be at least 5°C higher for the Grenoble area.

### Summer LWTs favorable to heatwaves and UHI

Extreme heat refers to both nocturnal UHI situations and biometeorological heatwaves. The different types of thermal extremes mentioned in this work are summarized in Table 4.

**Table 4:** Thermal extremes: definition and parameters.

Parameter	Maximal Temperature (Tx, in °C)	Minimal Temperature (Tn, in °C)	Duration
**Heatwave in the Isère Department**	Tx > 34 °C	Tn > 19 °C	3 consecutive days
**Biometeorological threshold for the Isère Department**	Tx > 34 °C	Tn > 19 °C	3 consecutive days
**Very hot day**	Tx ≥ 35 °C	–	–
**Hot day**	30 °C ≤ Tx < 35 °C	–	–
**Warm day**	25 °C ≤ Tx < 30 °C	–	–
**Cool day**	Tx < 25 °C	–	–
**Tropical Night**	–	Tn ≥ 20 °C	–
**UHI: Tn**_**urban**_ **-Tn**_**rural**_	dTn ≥ 3 °C and wind speed ≤ 2 m/s		

**Fig 8:**
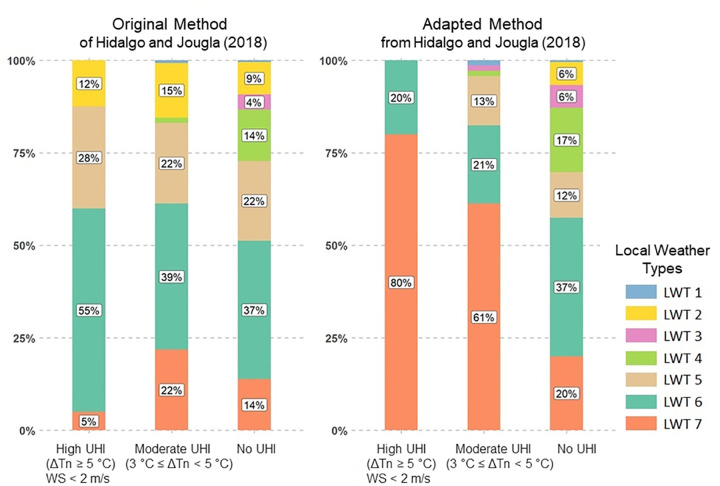
Comparison of UHI intensity in the Grenoble conurbation according to sensitive weather types, between the original method (from Hidalgo and Jougla, 2018) and the adapted one.

Fig 8 compares LWTs associated with different UHI intensities using the adapted method and the original method [47], with the same number of LWTs. The adapted method provides more distinct results for which LWTs represent UHI situations and hot days compared to the results from the original method applied the Grenoble region. Most of the days with high UHI (80%) and moderate UHI (61%) in summer are concentrated in LWT 7, whereas the results from the original method are scattered across various LWTs. This LWT 7 cluster is typical of summer days with UHI with high urban/rural thermal amplitude, low wind, absence of precipitation, and high specific humidity, *i.e.,* days of high dry heat.

**Fig 9:**
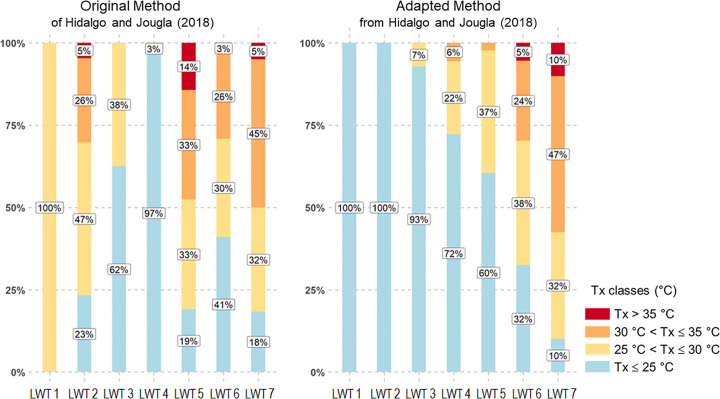
Comparison of four Tx classes in the Grenoble conurbation according to sensitive weather types, between the original method (from Hidalgo and Jougla, 2018) and the adapted one.

Moreover, Fig 9 details the distribution of maximum temperatures in four thermal classes within the seven LWTs, *i.e.,* cool days (Tx < 25 °C), heat days (25 °C < Tx < 30 °C), hot days (30 °C < Tx < 35 °C) and very hot days (Tx > 35 °C) (Table 4). LWTs 6 and 7 (adapted method) are the only ones to represent very hot days over the entire 2002–2022 period (respectively 5% and 10% of total days), in particular LWT 7.

**Fig 10:**
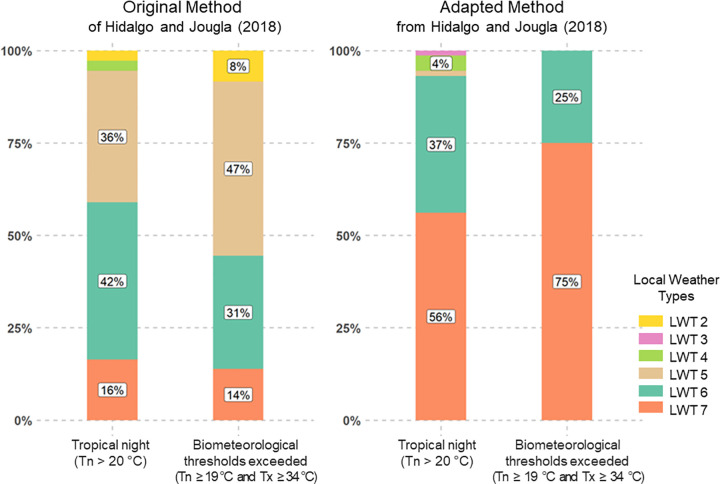
Tropical Nights and Heatwaves by Weather Type in Grenoble. Comparison of the occurrence of tropical nights and heatwaves – defined as exceedances of biometeorological thresholds – in the Grenoble conurbation across sensitive weather types, using both the original method of Hidalgo and Jougla (2018) and the adapted one.

Finally, Fig 10 shows the strong presence of tropical nights (Tn > 20°C) in the adapted method, concentrated in LWTs 7 and 6 (respectively 56 and 37% of days). Biometeorological heatwaves (locally, for the Isère department, Tn > 19 °C and Tx > 34 °C during three consecutive days) are found exclusively in situations associated with LWT 7 (75% of days) and LWT 6 (the remaining 25%); no heatwave is associated with any other types of atmospheric situations, which differs from the results of the original classification. LWT1 is not included here, as this type corresponds mainly to cold situations; tropical or warm nights in general are therefore not included.

## Discussion

**Fig 11:**
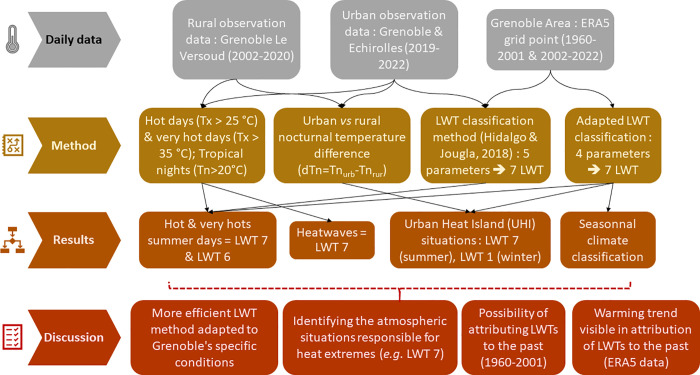
General methodology and main results of this study.

The methodological approach adopted in this paper is summarized in Fig 11, aiming to identify weather situations conducive to hot day types with or without pronounced UHI effects.

Between the 1951–1980 and 1991–2020 reference periods, Grenoble experienced a 1.5°C increase in annual mean temperature (10 °C to 11.5 °C), and 8% decrease in annual precipitation (993 mm ◊ 915 mm) at Saint-Geoirs station. This trend suggests a shift from temperate oceanic (Cfb) to humid subtropical (Cfa) climate, consistent with patterns observed in other French cities [66].

Although the emergence of hot summer months (> 22 °C) during 2021–2023 requires longer-term confirmation, it aligns with broader mid-latitude warming, particularly in Europe and the Alpine region [67].

ERA5 reanalysis offers global coverage and temporal consistency but may be biased in complex topography [62]. In Grenoble’s mountain valley, the spatial resolution smooths valley-floor conditions. We therefore evaluated ERA5 representativeness and integrated it with an adapted Local Weather Type (LWT) classification to identify local meteorological situations. In situ observations (2019–2022) provided essential information for UHI intensity, tropical nights, and ERA5 validation, reducing uncertainties without affecting LWT results.

The adapted LWT method [47] effectively distinguishes weather patterns in Grenoble’s complex topography. Among seven LWTs, LWT 7 -and to a lesser extent LWT°6- represents UHIs and hot days conditions. LWT 7 is characterized by dry, low-wind days with high diurnal temperature amplitude, favoring extreme heat events, including maximum temperatures above 35 °C and tropical nights (> 20 °C). ERA5 data reveal a rising trend in LWT 7 frequency since 1960, reflecting increased hot days and UHI occurrence, typically associated with westerly or southerly air masses.

A strong association exists between official heatwave alerts and UHI presence: 80% of Grenoble heatwave days and 100% in Échirolles coincide with UHI conditions. Conversely, 69.2% of Grenoble UHI days and 78.3% in Échirolles meet at least “normal” biometeorological thresholds.

Urban areas are highly vulnerable to extreme heat due to dense populations, infrastructure, and socio-economic exposure. Timely, precise heat alerts are essential, as the August 2003 heatwave tragically showed. The LWT method helps anticipate heat-related weather patterns, supporting urban risk management, city planning, emergency preparedness, and climate communication, with proven scientific credibility and versatility.

LWTs or other typologies are widely applied in urban climatology and adaptation studies, supporting urban planning, early warning systems, and climate communication [51,68–70]. Internationally, LWT-based analyses have been used to characterize UHI dynamics in France [71,72], Portugal [73], Poland [74], Greece [75], Spain [76], North Africa [70], the Middle East [77,78], and Canada [79], under humid, hot, or combined hot–humid conditions.

These studies consistently highlight weather types associated with extreme heat, enabling local authorities and decision-makers to implement targeted cooling strategies such as greening, depaving, water features, and reflective surfaces.

“Grenoble Alpes Métropole”, comprising 27 municipalities, pursues an ambitious climate adaptation and mitigation strategy. Originating from pioneering initiatives in Grenoble and Échirolles, these actions expanded across the metropolitan periphery. Measures include building retrofits, housing renovation, urban redevelopment, and green infrastructure preservation. The “Canopy Plan” supports evidence-based arboricultural management, while high-resolution urban heat island maps guide targeted interventions, such as schoolyard revegetation and cooling plans for vulnerable groups. These locally coordinated measures are aligned with France’s Third National Climate Change Adaptation Plan (PNACC 3), which provides the national framework for anticipating and mitigating climate impacts.

The refined LWT approach presented here, tailored to Grenoble’s topo-climatic context and limited to statistically robust weather types (RMSE < 0.05), enhances local diagnostic precision. It demonstrates the method’s adaptability to diverse geographic and climatic contexts and its value in supporting place-based adaptation strategies amid growing urban vulnerability and climate variability.

## Conclusion

This study provides a detailed characterization of the meteorological conditions driving extreme summer temperatures in Grenoble, a densely urbanized alpine valley subject to unique topo-climatic influences. We adapted the Local Weather Types (LWTs) classification [47], originally developed for flatter terrain, to account for the valley’s narrow floor and surrounding high mountains, enabling a precise assessment of local heat events.

Our analysis identified seven LWTs using Partitioning Around Medoids (PAM) on 20 years of daily meteorological data. In particular, LWT 7 is strongly linked to extreme summer events—heatwaves, tropical nights, and Urban Heat Island effects—and its increasing frequency reflects broader trends toward warmer, drier conditions. Wind absence, rather than direction, emerged as a key factor intensifying UHI phenomena in the valley.

The adapted LWT framework offers a flexible, reproducible tool for diagnosing urban climates, assessing long-term risks, and informing operational decision-making. Its successful application in Grenoble, combined with prior studies across Europe, North Africa, and the Middle East, demonstrates both its scientific robustness and global relevance for place-based adaptation strategies.
